# Mortalin deficiency suppresses fibrosis and induces apoptosis in keloid spheroids

**DOI:** 10.1038/s41598-017-13485-y

**Published:** 2017-10-11

**Authors:** Won Jai Lee, Hyo Min Ahn, Youjin Na, Renu Wadhwa, JinWoo Hong, Chae-Ok Yun

**Affiliations:** 1 Institute for Human Tissue Restoration, Department of Plastic & Reconstructive Surgery, Yonsei University College of Medicine, Seoul, Korea; 2 Department of Bioengineering, College of Engineering, Hanyang University, 222 Wangsimni-ro, Seongdong-gu, Seoul, 04763 Korea; 3 DAILAB, National Institute of Advanced Industrial Science and Technology (AIST), Central 5-41, 1-1-1 Higashi, Tsukuba, Ibaraki, 305-8565 Japan

## Abstract

Mortalin (Mot) is a mitochondrial chaperone of the heat shock protein 70 family and it’s pro-proliferative and anti-apoptosis functions could be associated with keloid pathogenesis, and blocking of mortalin and its interaction with p53 might be a potential novel target for the treatment of keloid. Therefore, we generated mortalin-specific small hairpin (sh) RNAs (dE1-RGD/GFP/shMot) and introduced into keloid spheroids for examination of its apoptotic and anti-fibrotic effect. On keloid tissues, mortalin expression was higher than adjacent normal tissues and it’s protein expressions were activated keloid fibroblasts (KFs). After primary keloid spheroid were transduced with dE1-RGD/GFP/shMot for knockdown of mortalin, expression of type I, III collagen, fibronectin, and elastin was significantly reduced and transforming growth factor-β1, epidermal growth factor receptor (EGFR), Extracellular Signal-Regulated Kinases 1 and 2 (Erk 1/2), and Smad 2/3 complex protein expression were decreased. In addition, increased TUNEL activities and cytochrome C were observed. Further, for examine of mortalin and p53 interaction, we performed immunofluorescence analysis. Knockdown of mortalin relocated p53 to the cell nucleus in primary keloid spheroids by dE1-RGD/GFP/shMot transduction. These results support the utility of knockdown of mortalin to induce apoptosis and reduce ECMs expression in keloid spheroid, which may be highly beneficial in treating keloids.

## Introduction

Keloids are defined as benign skin tumors, and occurred when the normal tissue repair sequence becomes dysregulated and the result of a prolonged proliferative and a delayed remodeling phase^1–3^. It caused by excessive extracellular matrix accumulation resulting from an aberrant extracellular matrix protein synthesis and degradation. Increased cell proliferation and an imbalance between collagen synthesis and degradation, accounting for the progressive and hypertrophic nature of keloids, correlates with reduced apoptosis, which may explain keloid pathogenesis^3–7^. Overexpression of tumor suppressor protein p53^7–9^ and mutation in the p53 gene^8–12^ had been found in keloids, and these may be linked to keloid pathogenesis. p53 is a nuclear transcription factor often found in cytosol which translocates to the nucleus in response to stress, ultimately promoting apoptosis of damaged cells^3,6,13,14^.

Mortalin (Mot; mtHsp70/PBP74/Grp75) is a 679 amino acids long (MW 73,913 Da) heat un-inducible member of Hsp70 family of proteins and plays an essential role in mitochondrial import, oxidative stress response, regulation of mitochondrial membrane potential, energy generation, intracellular transport, chaperonization, protection against apoptosis, and p53 functions^15–17^. Mortalin and p53 interactions were first identified in the cytoplasm of tumor cells. The pro-proliferative effects of mortalin overexpression in cancer cells have been assigned to its binding with p53 that results in its retention in the cytoplasm, and inhibition of its normal transcriptional activation function of p53^16,18–21^, resulting in lifespan extension of cells, uncontrolled proliferation, and malignant transformation. But in normal human cells, mortalin predominantly present in mitochondria where it mediates transcription-independent tumor suppression by induction of mitochondrial permeabilization and apoptosis^22,23^. Several other studies have assigned in line with an anti-apoptotic function to mortalin^21,23–26^. Anti-mortalin molecule, such as antisense, ribozyme, and shRNA that that abrogated mortalin-p53 interaction and caused the relocation of p53 to the cell nucleus, resulted in growth arrest/apoptosis of cancer cells^19,26–28^. However, the expression of mortalin and mortalin-p53 interaction on the keloid was not investigated.

Keloid scars are locally aggressive, continually grow, and invade the surrounding normal skin, and are caused by increased proliferation and an excess collagen deposition by abnormal fibroblasts. On the basis of these characteristics of keloid, we hypothesize that the pro-proliferative and anti-apoptosis functions of mortalin could be associated with keloid pathogenesis, and targeting mortalin and its interaction with p53 might be a potential novel target for the treatment of keloid. Therefore, in the present study, we investigate the expression of mortalin in keloid and normal tissues, where it protects cell apoptosis by mechanisms involving inactivation of p53 functions. Also, we generated mortalin-specific small hairpin (sh)RNAs (dE1-RGD/GFP/shMot) and introduced into keloid spheroids for examination of its apoptotic and anti-fibrotic effect.

## Results

### Mortalin expression was increased in keloid tissues compared with adjacent normal tissues

**Figure 1:**
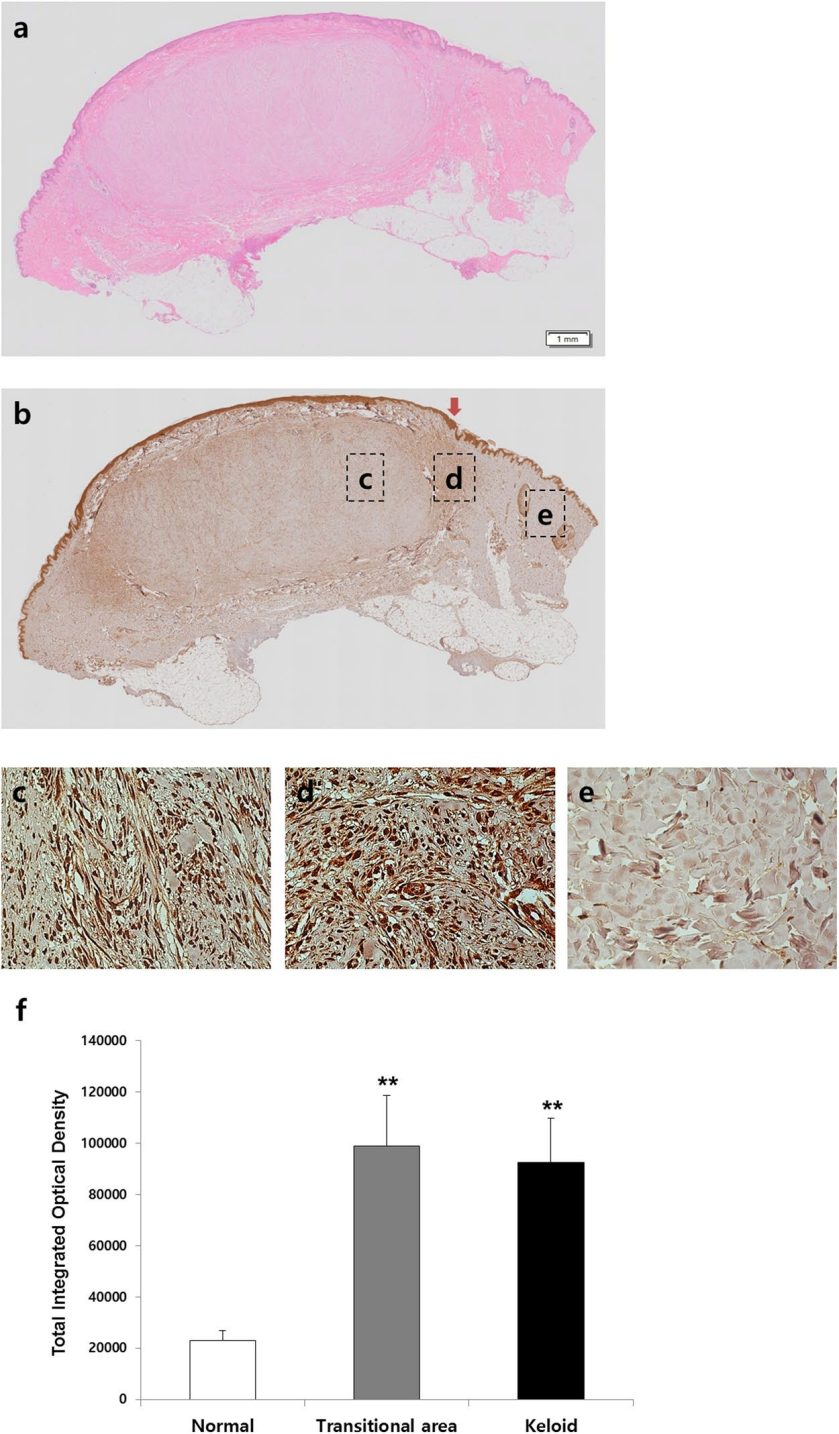
Immunohistochemical staining of mortalin in human keloid tissues. (**a**) Keloid tissue had a dense and excessive collagen deposition that extended over the clinical keloid margin into the extra-lesional dermal tissue. The expression of mortalin in keloid tissue (**b** & **d**) was increased than that in adjacent normal tissue (e), especially peripheral keloid region (**d**). (**f**) On the semi-quantitative Metamorph^®^ image analysis, the expression of mortalin increased by 4.8 times than adjacent normal tissue, and this difference was statistically significant (**p < 0.01).

After hematoxylin and eosin (H&E) staining, we observed that keloid tissue had a dense and excessive collagen deposition that extended over the clinical keloid margin into the extra-lesional dermal tissue (Fig. 1a). To evaluate mortalin protein expression patterns in keloid tissue, immunohistochemical staining was performed (n = 5). Compared to extra-lesional normal tissue (Fig. 1b & e), markedly increased mortalin immunoreactivity was noted in central and peripheral keloid region (Fig. 1c & d). The increased expression of mortalin was semi-quantitatively measured with MetaMorph® image analysis software (Fig. 1f). The results showed that mortalin protein levels were markedly higher in the central and transitional regions of the keloids (optical density; 92446 ± 17322, 99007 ± 19811, respectively) than in normal tissue (optical density; 23005 ± 3969). Protein expression levels of mortalin in keloid tissues were 4.8 times that of normal tissue (**p < 0.01).

**Figure 2:**
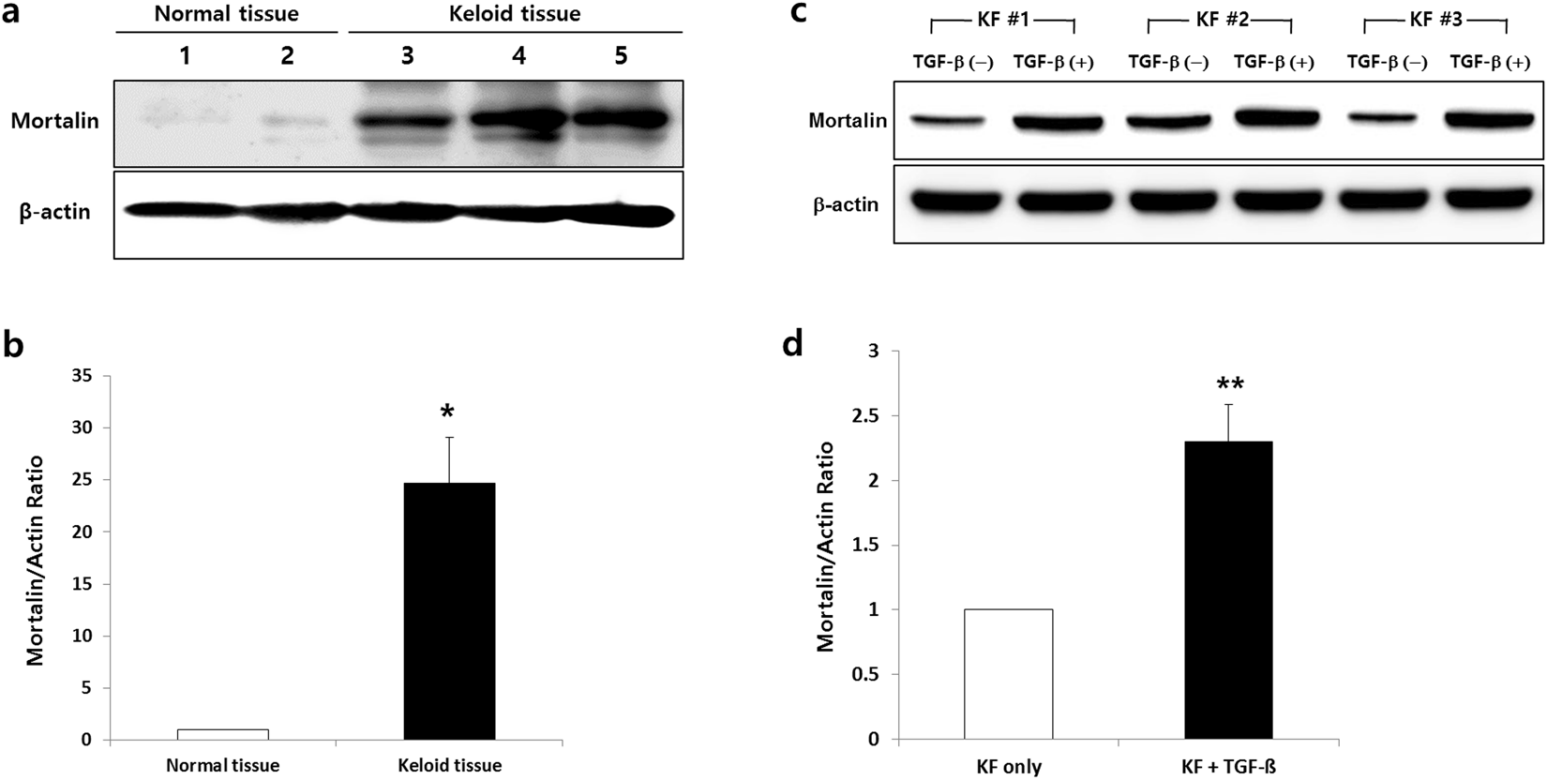
Expression of mortalin protein in keloid tissues by western blot. (**a** & **b**) The expression of mortalin protein was increased by 25 times on the keloid tissues compared to normal dermal tissues. (**c** & **s**) Also, mortalin protein expressions were more increased by 2.3 times on the activated KFs by TGF-β1 (10 ng/mL).

Mortalin protein expression was investigated with Western blot analysis (n = 3). As shown in Fig. 2, mortalin protein levels were 25 times higher in KFs relative to normal dermal tissue, which was further upregulated by 2.3-fold when KFs were activated with transforming growth factor (TGF)-β1 (10 ng/mL), a strong fibrosis inducing factor that plays an important role in the keloid pathophysiology.

### Construction of mortalin specific shRNA-expressing adenovirus and their effect on the KFs and keloid spheroids

**Figure 3:**
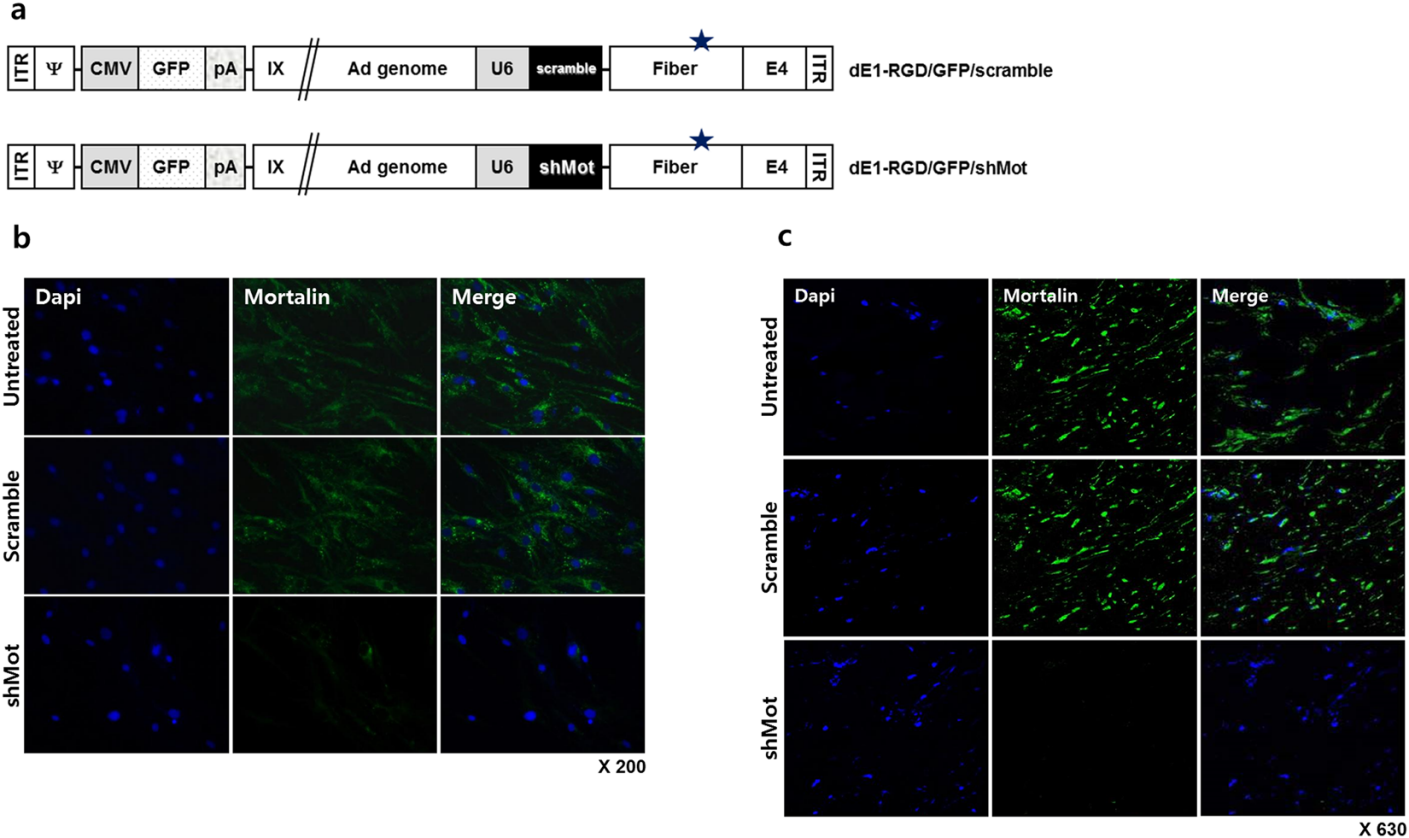
Construction of shMot-expressing adenovirus. (**a**) Schematic representation of the shMot-expressing adenoviral vectors. The RGD-incorporated adenovirus was generated by inserting RGD motif between HI-loop of the fiber knob (star). (ITR = inverted terminal repeat; Ψ = packaging signal; pA = polyA sequence; IX = protein IX; and shMot = mortalin-specific small hairpin (sh)RNAs). (**b**) Mortalin expression in keloid fibroblasts (KFs) after transduction with dE1-RGD/GFP/shMot. Scrambled shRNA not targeting any known human gene was used as a control. Overexpressed mortalin on the cytosolic and extracellular area of KFs, which were examined by immunofluorescence assay, were extremely knockdown by the treatment of dE1-RGD/GFP/shMot. Double immunostaining images of KFs show mortalin knockdown (mortalin, green; nucleus, blue; ×200). (**c**) The similar results were obtained on the primary keloid spheroids after infection of dE1-RGD/GFP/shMot (x680).

On the basis of the results that mortalin expression increased on the keloid tissues, we anticipated that inhibition of mortalin could be applied for keloid or hypertrophic scar treatment. Therefore, we generated mortalin-specific shRNA-expressing adenovirus (Fig. 3a) and examined their effect on KFs and keloid spheroids. As shown in Fig. 3b and c, overexpressed mortalin on the cytosolic and extracellular area of KFs (Fig. 3b) and primary keloid spheroid (Fig. 3c), which were examined by immunofluorescence assay, were extremely knockdown by the treatment of dE1-RGD/GFP/shMot.

### Mortalin specific shRNA-expressing adenovirus decreases collagen type I and III, elastin, and fibronectin protein expression in primary human keloid spheroids

**Figure 4:**
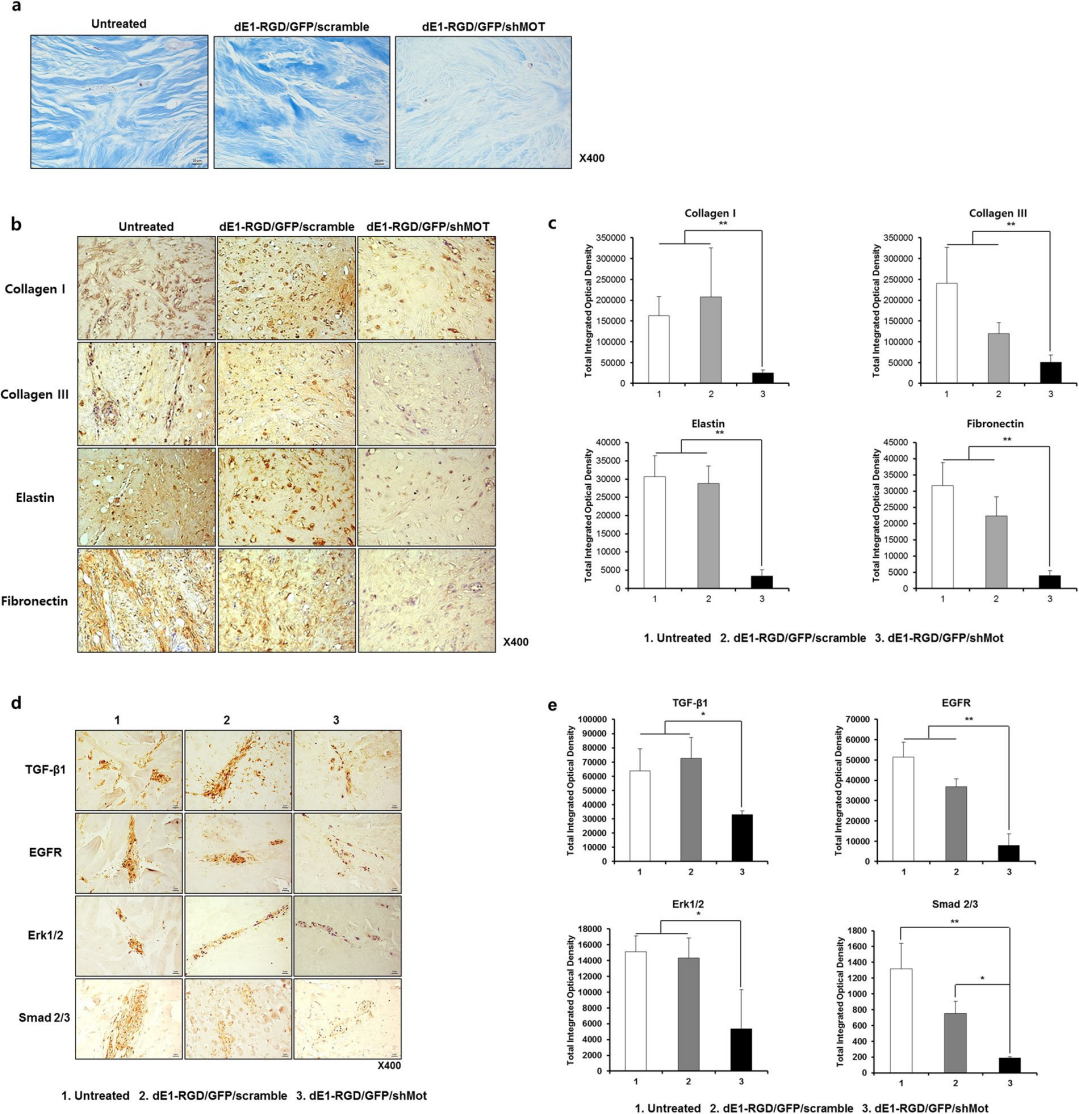
Immunohistochemical staining of type I and III collagen, elastin, and fibronectin protein from dE1-RGD/GFP/shMot-transduced keloid spheroid tissues. (**a**) Masson’s trichrome staining of keloid spheroids. After dEl-RGD/GFP/shMot treatment of keloid spheroids, dense and coarse collagen bundles were replaced with thin and shallow collagen bundles. Original magnification: x400. (**b**) Reduced expression of ECM components including collagen type I and III, elastin, and fibronectin protein in keloid spheroids transduced with dE1-RGD/GFP/shMot compared to those in spheroids transduced with scramble virus. Original magnification: 400x. (**c**) Semi-quantitative image analysis for type I and III collagen, elastin, and fibronectin protein expression. Significant reduction in type I collagen, type III collagen, elastin, and fibronectin was observed in keloid spheroids transduced with dE1-RGD/GFP/shMot in comparison to untreated group or scramble virus-treated virus (**p < 0.01). (**d**) Reduced expression of TGF-β1, EGFR, Smad 2/3 complex, and Erk 1/2 protein in primary keloid spheroids by mortalin specific shRNA-expressing adenovirus (x400). (**e**) The decreased expression of TGF-β1, EGFR, Smad 2/3 complex, and Erk 1/2 were observed in mortalin specific shRNA-expressing adenovirus treated keloid spheroids, by 52%, 43%, 11%, and 42%, respectively, versus scramble virus-transduced spheroids (*p < 0.05, **p < 0.01).

Keloid spheroids derived from active-stage keloid patients (n = 3) were cultured and transduced with either dE1-RGD/GFP/scramble or dEl-RGD/GFP/shMot. The effect of mortalin on expression of major ECM components of keloid was evaluated histologically. Masson’s trichrome of keloid sections revealed that collagen deposition was decreased in spheroids transduced with dEl-RGD/GFP/shMot versus scramble virus (Fig. 4a). In addition, dense and coarse collagen bundles structure were replaced by thin and shallow collagen bundles. Image analysis of immunohistochemical staining also revealed significantly reduced type I collagen, type III collagen, elastin, and fibronectin in dEl-RGD/GFP/shMot treated keloid spheroids, by 12%, 43%, 12%, and 18%, respectively, versus scramble virus-transduced spheroids (**p < 0.01 in all cases; Fig. 4b and c). Taken together, these data strongly suggest that expressions of the major ECM components such as collagen type I and III, elastin, and fibronectin are significantly decreased by knockdown of mortalin expression in primary keloid spheroid.

### Mortalin specific shRNA-expressing adenovirus elicited decreased expression of TGF-β1, EGFR, and Erk 1/2 on keloid spheroids

We examined that dEl-RGD/GFP/shMot may be decreased expression of TGF-β1 and EGFR on keloid spheroid compared with dE1-RGD/GFP/scramble. Also, to examine the intracellular effect of mortalin specific shRNA-expressing adenovirus, Smad 2/3 complex and Erk 1/2 protein expressions were investigated. As shown in Fig. 4d, significantly decreased expressions of TGF-β1, EGFR, Smad 2/3 complex, and Erk 1/2 protein were observed in mortalin specific shRNA-expressing adenovirus treated keloid spheroids, by 52%, 43%, 11%, and 42%, respectively, versus scramble virus-transduced spheroids (*p < 0.05, **p < 0.01; Fig. 4d and e). These results demonstrated that knockdown of mortain can attenuate EGF/EGFR signaling pathway and TGF-β1/Smad pathway, which have an important role in the fibrogenesis. Also, overexpressed mortalin has an important role in keloid pathogenesis and inhibition of mortalin expression could be a therapeutic target on the treatment of keloid or hypertrophic scar.

### Knockdown of mortalin decreased PCNA expression and induced apoptosis

**Figure 5:**
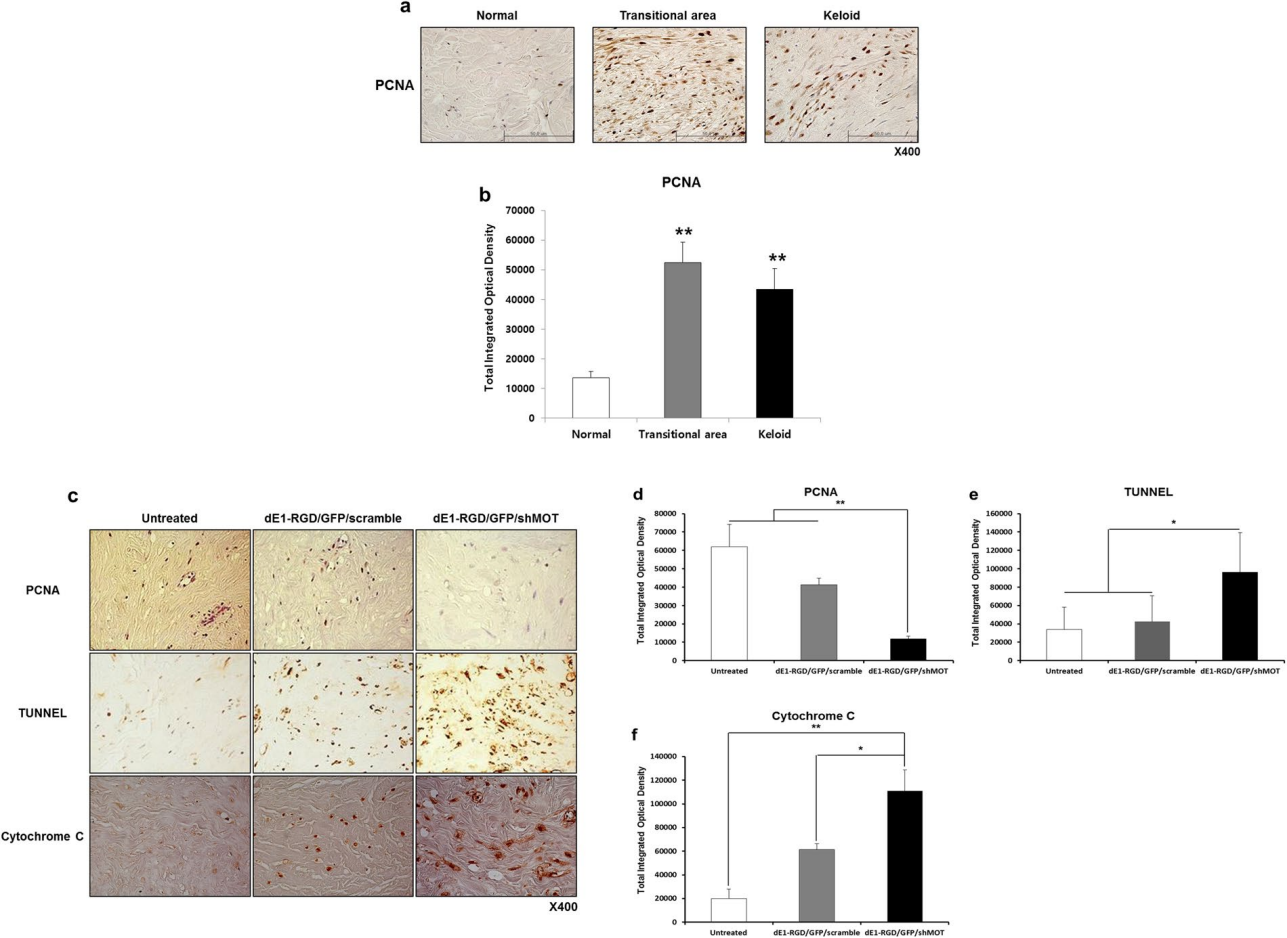
Knockdown of mortalin decreased PCNA expression and induced apoptosis. (**a**) Proliferating cell nuclear antigen (PCNA) immunohistochemical staining on keloid tissues (n = 3). (b) Expression of PCNA protein markedly increased in central and transitional keloid region compared to adjacent normal tissue by 3.9 times and 3.2 times (**p < 0.01). (**c**–**e**) Reduced expression of PCNA (**c** & **d**) and a marked increase in apoptosis as observed by TUNEL staining on the dEl-RGD/GFP/shMot treated keloid spheroids (**c** & **e**), and expressions of cytochrome C protein was increased by 4.4- and 1.8-fold after dEl-RGD/GFP/shMot transduction in comparison to untreated or scramble virus treated spheroid (**c** & **f**) (*p < 0.05; **p < 0.01). Original magnification: x400.

Keloid formation is often considered to be the result of a prolonged cellular proliferation and reduced apoptosis. Therefore, proliferative activities of keloid was examined by a proliferating cell nuclear antigen (PCNA) immunohistochemical staining on keloid tissues (n = 5), resulted that expression of PCNA protein markedly increased in central and transitional keloid region compared to adjacent normal tissue by 3.9 times and 3.2 times, respectively (Fig. 5a and b). Next, we examined whether the knockdown of mortalin could decrease cellular proliferation and induce apoptosis in scramble and mortalin specific shRNA-expressing adenovirus-transfected keloid spheroid. Image analysis of immunohistochemical staining revealed significantly reduced PCNA expression in dEl-RGD/GFP/shMot treated keloid spheroids by 29% (Fig. 5c and d).

Further, to determine whether reduced mortalin expression induced apoptosis, a TUNEL assay and cytochrome C immune-staining were carried out using the keloid spheroid. As shown in Fig. 5c and d, TUNEL positivities were increased on the dEl-RGD/GFP/shMot-treated keloid spheroids, and expression of cytochrome C protein was increased by 1.8 times after dEl-RGD/GFP/shMot transduction. In addition, wild type p53, phospho-p53, p21, and caspase 3 were increased by 2.8-, 8.5-, 3.2-, and 5.7-fold, respectively, in primary keloid fibroblast after transduction with dE1-RGD/GFP/shMot (200 MOI) in comparison with dE1-RGD/GFP/scramble (200 MOI) group (Supplementary Fig. S2). Taken collectively, our results suggest that knockdown of mortalin protein decrease cell proliferation and induce apoptosis, and mortalin may be a target for keloid treatment.

### Mortalin as p53 inactivator; Mortalin interacts with p53 and its knockdown relocate p53 to the cell nucleus in primary keloid spheroids

**Figure 6:**
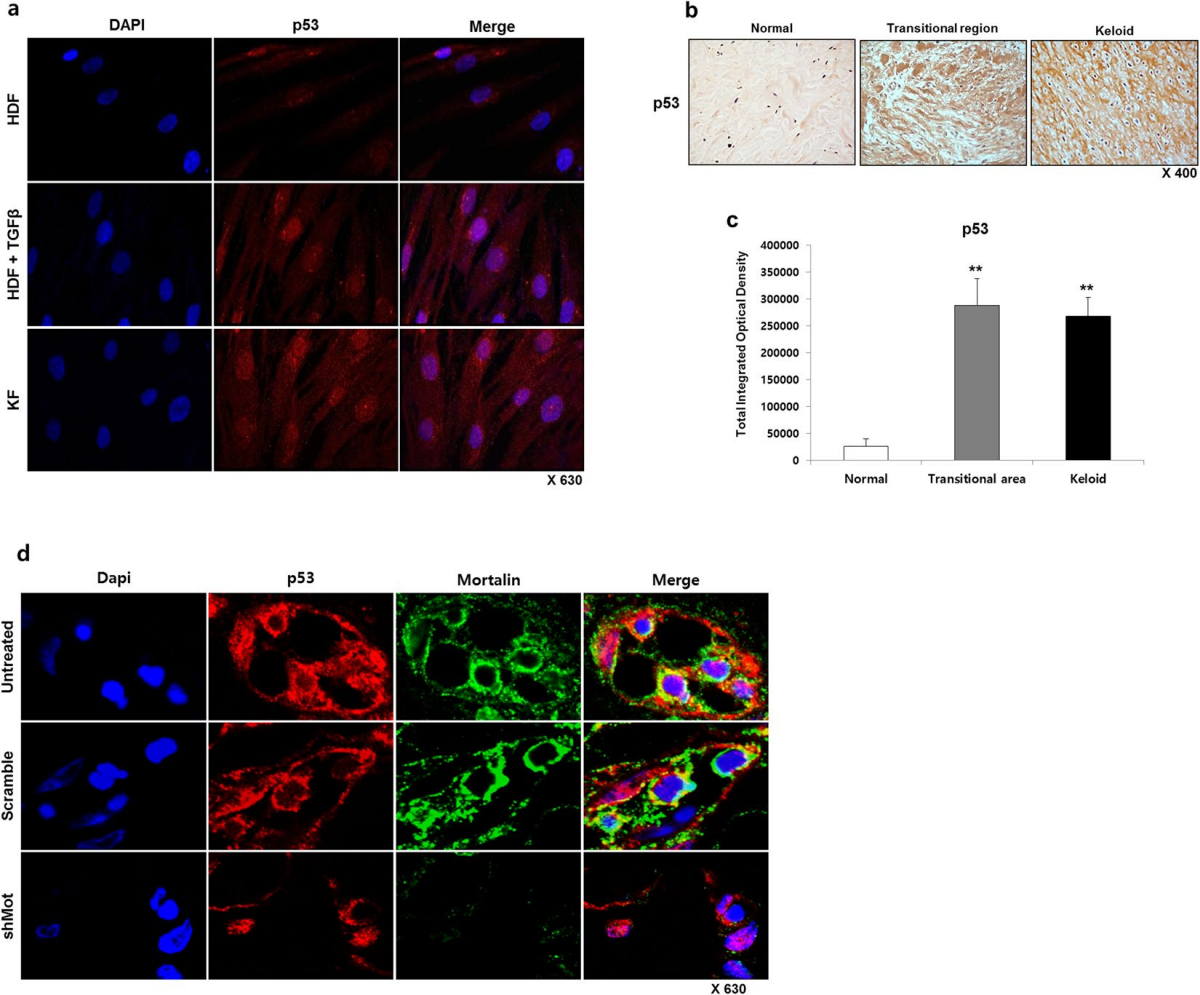
Mortalin interacts with p53 and its knockdown relocates p53 to the cell nucleus in primary keloid spheroids. (**a**) p53 immunofluorescence staining of HDFs, TGF-β1-activated HDFs, and KFs. A clear and strong localization of p53 is shown from the cytoplasm and nucleus of TGF-β1-activated HDFs and KFs, compared to its HDFs (p53, red; nucleus, blue; x680). (**b**–**c**) Expression of p53 protein markedly increased in central and transitional keloid region compared to adjacent normal tissue by approximately 10 times (n = 6) (**p < 0.01). (**d**) Mortalin silencing-induced apoptosis is mediated by nuclear translocation of mutant p53. Colocalizations of p53 and mortalin in the cytosol were investigated on the untreated keloid spheroid. Double immunostaining images of mortalin specific shRNA-expressing adenovirus treated keloid spheroids show mortalin knockdown-induced nuclear translocation of p53 (mortalin, green; p53, red; DAPI, blue).

As shown in Fig. 6, overexpression of p53 protein on the KFs (Fig. 6a) and keloid tissues (n = 5) (Fig. 6b-c) were confirmed, and the localization of p53 was found in the cytoplasm as well as the nucleus, but most of them expressed in the cytoplasm. The cytoplasmic accumulation of p53 was especially noticeable on the keloid tissues. We next investigated whether mortalin would cause inactivation of p53 function by cytoplasmic sequestration. If so, apoptosis caused by mortalin knockdown would be expected to involve nuclear translocation of the p53 and induce an apoptosis by reactivation of p53. To test this hypothesis, we performed immunofluorescence analysis on keloid spheroid (n = 3). Transduction of dEl-RGD/GFP/shMot led to significant accumulation of p53 in the nucleus. As shown in Fig. 6d, colocalizations of p53 and mortalin in the cytosol were investigated on the untreated keloid spheroid. After mortalin specific shRNA-expressing adenovirus was treated on the keloid spheroids, mortalin expressions were knockdown and they was exhibited intense nuclear staining for p53, suggesting the nuclear translocation. In line with these findings, a time-dependent increase in nuclear p53 level was confirmed by western blot analysis in dEl-RGD/shMot-treated primary keloid fibroblast (Supplementary Fig. S3). In contrast, no nuclear p53 level was increased in scrambled shRNA-expressing Ad-treated cells.

## Disscusion

Keloids are fibro-proliferative skin lesions that are characterized by excessive accumulation of ECM components–mainly collagens–and invasion into normal peripheral regions. Increased cell proliferation, accounting for the progressive and hypertrophic nature of keloids, correlates with a lower rate of apoptosis^5,7,9^, which may play a role in the process of pathological scarring. Therefore, apart from the researches keloid pathogenesis caused by increased proliferation and an excess collagen deposition by fibroblasts, the understanding of the mechanisms by which keloids escape from apoptosis could be beneficial in the development of novel therapeutic strategies.

Our data showed that mortalin and tumor suppressor protein p53 expression were increased on the keloid tissues compared with adjacent normal tissues, especially on the cytosol. In addition, mortalin expression significantly increased after TGF-β1 (10 ng/mL) treatment on keloid fibroblasts. Mortalin (mot-2/mthsp70/PBP74/GRP75) is an essential protein belonging to the Hsp70 family of chaperones, and its overexpression confers proliferation-growth advantages to cells^15,16,18,20,21^. Mortalin is primarily localized in the mitochondria but is also found in the endoplasmic reticulum, plasma membrane, and cytoplasmic vesicle^29^. It is also found as a secreted protein and detected in the extra-cellular space^29^. It protects cells from senescence and apoptosis and is overexpressed in cancer cells, but yet few report identified its role in fibrotic disease such as keloid. Therefore, we think that overexpressed mortalin in keloid tissue compared to the adjacent normal tissue could be an important role in fibrogenesis. In light of this, we generated mortalin-specific small hairpin (sh)RNAs (dE1-RGD/GFP/shMot) and introduced into keloid spheroids for examination of its apoptotic and anti-fibrotic effect. After the transduction of dE1-RGD/GFP/shMot, expressions of the major ECM components are significantly decreased in primary keloid spheroid, and knockdown of mortain can attenuate EGF/EGFR signaling pathway and TGF-β1/Smad pathway, which have a role in the fibrogenesis. The number of studies revealed that extracellular Hsp70 (Hsp 72) promotes TLR4 signaling leading to TGF-β1 activation^30–33^, and Hsp47 is a collagen-specific molecular chaperone that plays a critical role in normal procollagen biosynthesis in mammals^34,35^. However, the functional rule of mortalin for the fibrogenesis remains unclear. Therefore, additional researches will be needed.

The fibrotic process is maintained by the inhibition of apoptosis in myofibroblasts, which produce an excess of ECM, thereby causing keloid or hypertrophic scar. A number of nuclear proteins have been shown to redistribute to the cytosol and mitochondria at the onset of apoptosis. Among these, the tumor-suppressor protein p53 is a short lived nuclear phosphoprotein, which has transcription-dependent effects that involves induction of pro-apoptotic genes^14,36,37^ and overexpression of p53^7–9^ had been found in keloids. In line with these reports, primary patient keloid fibroblast samples used in this study expressed wild type p53 (Supplementary Fig. S1).

The effects of overexpressed mortalin such as malignant transformation^38^, life span extension^15^, and attenuation of differentiation^39^ has been attributed, at least in part, to transcriptional inactivation of p53. There is well known that mortalin (amino-terminus region) was found to bind to the carboxy-terminus region of p53, resulting in cytoplasmic retention and transcriptional inactivation of p53^16,18–21^. Also, it localizes in the nucleus, inactivates p53-mediated apoptosis^28^. On the basis of theses knowledge, we blocked the cytosolic mortalin by dE1-RGD/GFP/shMot, and expected it to discharge p53 from mortalin–p53 complex resulting in its functional activation. As shown in our data, we examined that knockdown of mortalin induced nuclear translocation of the p53 and decrease cell proliferation. Also, apoptotic activity, cytochrome C release, and TUNEL positivities were increased on the dE1-RGD/GFP/shMot treated keloid spheroid.

On our researches, we found that functional roles of dE1-RGD/GFP/shMot such as anti-proliferative function, antifibrosis, and increase apoptotic activity could be expected to be of great advantage in keloid or hypertrophic scars therapeutics. Despite this role, the potential therapeutic use of shMot-expressing adenovirus possesses limitations, such as a transient effect, which is not desirable to chronically persistent keloids, acute inflammatory responses, and the innate immune response. Exhaustive research efforts has prompted the development of novel strategies to overcome these limitations^40^. Therefore, we think that further refinement studies will be needed for more efficient and safe gene transfer in patients.

## Materials and Method

### Human dermal fibroblast, keloid tissue, and keloid-derived fibroblast cells

Human normal dermal fibroblasts (HDFs) and keloid fibroblasts (KFs) were obtained from the ATCC (American Type Culture Collection, Manassas, VA). After obtaining informed consent according to a protocol approved by the Yonsei University College of Medicine Institutional Review Board, keloid tissues and normal abdominal skin tissues were obtained for fibroblast culture, histologic, and immunohistochemical analysis with excision. Keloid fibroblasts were obtained from both the central dermal layer of keloids (Supplementary Table 1). All experiments involving humans were performed in adherence to the Helsinki Guidelines. Cells were cultured in Dulbecco’s Modified Eagle’s Medium (DMEM; GIBCO, Grand Island, NY) supplemented with 10% heat-inactivated fetal bovine serum (FBS), penicillin (100 U/mL), streptomycin (100 μg/mL).

### Immunohistochemistry (IHC) for mortalin, p53, and PCNA

Formaldehyde-fixed tissues were transferred to a paraffin-embedded block, sectioned at 4-μm thicknesses. After tissue deparaffinization and rehydration, endogenous peroxidase activity was blocked by 10-min incubation at room temperature with absolute methanol containing 1% hydrogen peroxide. The tissue sections were incubated with a primary antibody against mouse anti-mortalin monoclonal antibody (C1-3), rabbit anti-p53 (sc-6243; Santa Cruz biotechnology, Santa Cruz, CA), and mouse anti-PCNA (M0879; DAKO, Carpinteria, CA) at 4 °C overnight. After incubation with the secondary antibody (Super Sensitive™ Polymer-HRP IHC, BioGenex) for 1 h at room temperature, the bound complexes were visualized by incubating tissue sections with 0.05% diaminobenzidine and 0.003% hydrogen peroxide. The sections were counterstained with Harris hematoxylin and then dehydrated and mounted. Mortalin, p53, and PCNA protein levels were semi-quantitatively analyzed using MetaMorph^®^ image analysis software (Universal Image Corp., Buckinghamshire, UK). Results are expressed as mean optical density of six different digital images per sample.

### Generating shMot-expressing adenoviral vectors

A replication-incompetent Ad expressing shMot (dE1-RGD/GFP/shMot; Ad-shMot) and control Ad (dE1-RGD/GFP/scramble; Ad-scramble) were used in this study (Fig. 3). Both replication-incompetent Ads possess RGD, a tripeptide composed of L-arginine, glycine, and L-aspartic acid that recognizes subtypes of integrins, in their fiber to improve the transduction to keloid fibroblasts in comparison to wild-type Ad by providing an alternative viral entry pathway into keloid fibroblasts^41,42^. To generate an Ad expressing GFP and shMot or scramble at the E1 and E3 regions, respectively, pdE1-RGD/GFP^43^ was linearized by *Spe*I digestion and co-transformed into *Escherichia coli* BJ5183 with the *XmnI*-digested pSP72-E3/CMV-shMot or -scramble E3 shuttle vector^44^ for homologous recombination, generating a pdE1-RGD/GFP/shMot or /scramble adenoviral vector. The propagation, purification, and titration of Ad were performed as described previously^45,46^.

### Preparation and adenoviral transduction of keloid spheroids

Keloid tissues were obtained from active-stage keloid patients (n = 5). Keloid spheroids were prepared by dissecting keloid central dermal tissue into 2-mm diameter pieces with sterile 21-gauge needles. Explants were plated onto HydroCell^®^ 24 Multi-well plates (Nunc, Rochester, NY) after which they were cultured for 4 h in IMDM (Isocove’s modified Dulbecco’s medium, Gibco BRL) supplemented with 5% fetal bovine serum, 10 mM l^−1^ insulin and 1 mM l^−1^ hydrocortisone. Each of the Ads (dE1-RGD/GFP/shMot and dE1-RGD/GFP/scramble) at 1 × 10^10^ VP were added into the plates containing keloid spheroid, and incubated at 37 °C in 5% CO_2_ incubator for 3 days. The transduced keloid spheroids were then fixed with 10% formalin, paraffin-embedded, and cut into 5-μm-thick sections.

### Histology and immunohistochemistry

Representative sections were stained with hematoxylin and eosin (H & E) and Masson’s trichrome, and then examined by light microscopy. Keloid spheroid sections were incubated at 4 °C overnight with mouse anti-collagen type I (ab6308; Abcam, Ltd., Cambridge, UK), mouse anti-collagen type III (C7805; Sigma, St. Louis, MO), mouse anti-elastin (E4013; Sigma), mouse anti-fibronectin (sc-52331; Santa Cruz Biotechnology), rabbit anti-TGF-β1 (ab9758; Abcam, Cambridge, UK), mouse anti-EGFR (Ab-1; Oncogene Research Products, Calbiochem), rabbit anti-Erk 1/2 (#4370 S; Cell Signaling Technology, Beverly, MA), and rabbit anti-Smad 2/3 complex (#8685 S; Cell Signaling Technology) mouse anti-PCNA (DAKO), goat anti-Cytochrome c (SC-8385; Santa Cruz Biotechnology) primary antibody, and then incubated at room temperature for 20 min with the Dako Envision™ Kit (DAKO, Glostrup, Denmark) as secondary antibody. Diaminobenzidine/hydrogen peroxidase (DAKO, Carpinteria, CA) was used as the chromogen substrate. All slides were counterstained with Meyer’s hematoxylin. The expression levels of TGF-β1, EGFR, Erk 1/2, Smad 2/3, type I and III collagen, elastin, and fibronectin were semi-quantitatively analyzed using MetaMorph^®^ image analysis software (Universal Image Corp., Buckinghamshire, UK). Results are expressed as the mean optical density for six different digital images.

### Western blot analysis

KFs were lysed in a solution containing 50 mM Tris-HCl (pH 7.6), 1% Nonidet P-40 (NP-40), 150 mM NaCl, 0.1 mM zinc acetate, and protease inhibitors. Protein concentration was determined by the Lowry method (Bio-Rad, Hercules, CA), and 30 μg of each sample was separated by sodium dodecyl sulfate-polyacrylamide gel electrophoresis (SDS-PAGE). The proteins on the gel were electrotransferred to polyvinylidene fluoride membrane, incubated with the primary mouse anti-mortalin monoclonal antibody (C1-3) and rabbit anti-β-actin antibody (Sigma, St Louis, MO), and then secondarily incubated with the HRP (horseradish peroxidase)-conjugated secondary antibody (#7074 or 7076; Cell Signaling Technology). The expression patterns were revealed using the ECL detection kit (sc-2004; Santa Cruz Biotechnology), and the expression levels of mortalin was developed using enhanced chemiluminescence (Amersham Pharmacia Biotech, Uppsala, Sweden). Mortalin protein was semi-quantitatively analyzed using ImageJ software (National Institutes of Health, Bethesda, MD).

### Immunofluorescence assay

Keloid tissue sections (n = 4) were deparaffinized, rehydrated, blocked with 5% goat serum, and incubated with mouse anti-mortalin monoclonal antibody (C1-3) or rabbit anti-p53 (sc-6243; Santa Cruz biotechnology) primary antibody overnight at 4 °C. Day after incubation, sections were washed with phosphate-buffered saline (PBS) and incubated with Alexa Flour 488-conjugated goat anti-mouse IgG (A11001; Invitrogen, Carlsbad, CA) or Alexa Flour 633-conjugated goat anti-rabbit IgG (A21070; Invitrogen) secondary antibody for 60 min at room temperature. Tissues were mounted on slides using Vectashield^®^ mounting medium containing the nuclear stain DAPI (Vector Laboratories, Burlingame, CA), and viewed by confocal microscopy (LSM700, Olympus, Center Valley, PA).

### Terminal Deoxynucleotidyl Transferase dUTP Nick End Labeling (TUNEL) assay

The apoptotic spheroid population was assessed by TUNEL assay as described previously (Gene Ther, 2008. **15**(9): p. 635-51). The apoptotic cells were visually identified in five randomly selected fields and photographed at a magnification of x100 and x400. The expression level of apoptotic spheroid was semi-quantitatively analyzed using MetaMorph^®^ image analysis software. Results are expressed as the mean optical density for five different digital images.

### Statistics

Results are expressed as the mean ± standard error of the mean (SEM). Data were analyzed by a repeated-measures one-way ANOVA. Two sets of independent sample data were compared using a paired t-test; *p*-values < 0.05 was considered indicative of statistically significant differences.

## Electronic supplementary material

Supplementary Figure Legends
