# Supplementary material for: Mortalin deficiency suppresses fibrosis and induces apoptosis in keloid spheroids
Source: Sci Rep. 2017 Oct 11;7:12957. doi: 10.1038/s41598-017-13485-y (PMC5636810; doi:10.1038/s41598-017-13485-y)

Mortalin deficiency suppresses fibrosis and induces apoptosis in keloid spheroids

Won Jai Lee[1], Hyo Min Ahn[2], Youjin Na[2], Renu Wadhwa[3], JinWoo Hong[2], Chae-Ok Yun[2]*

[1]Institute for Human Tissue Restoration, Department of Plastic & Reconstructive Surgery, Yonsei University College of Medicine, Seoul, Korea, [2]Department of Bioengineering, College of Engineering, Hanyang University, 222 Wangsimni-ro, Seongdong-gu, Seoul 133-791, Korea, [3]DAILAB, National Institute of Advanced Industrial Science and Technology (AIST), Central 5-41, 1-1-1 Higashi, Tsukuba, Ibaraki, 305-8565, Japan.

Supplementary Table 1. Demographic information and description of keloids obtained from study subjects

Case (no.)	Sex	Age (years)	Keloid site	Used for
K1	M	31	Chest	IHC
K2	F	33	Ear lobe	IHC
K3	F	11	Knee	IHC, spheroid
K4	F	50	Anterior chest	IHC
K5	F	5	Ankle	IHC
K6	F	18	Earlobe	WB
K7	F	17	Earlobe	WB, spheroid, IHC
K8	M	53	Shoulder	WB, spheroid Immunofluorescence
K9	F	18	Earlobe	Immunofluorescence
K10	F	26	Earlobe	Immunofluorescence
K11	F	24	Earlobe	Immunofluorescence Spheroid, IHC
K12	F	33	Abdomen	Immunofluorescence Spheroid, IHC
K13	M	4	Neck	Spheroid, IHC

IHC, immunohistochemistry; WB, western blotting.

Supplementary Figure Legends

Supplementary Figure S1. Immunoblotting analysis was performed to analyze the expression levels of wild type p53 in several primary keloid fibroblasts (Patients K6, K7, K8).

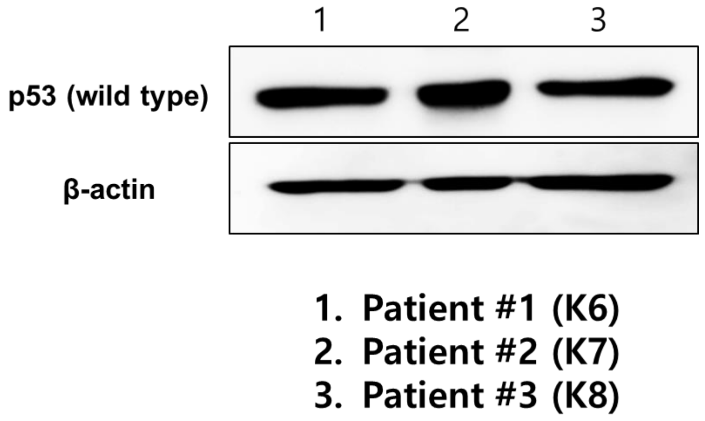

Supplementary Figure S2. Immunoblotting analysis was performed to analyze the expression levels of wild type p53, phospho-p53, p21, and caspase 3 in primary keloid fibroblast after transduction with either dE1-RGD/GFP/scramble or dE1-RGD/GFP/shMot (100 and 200 MOI).

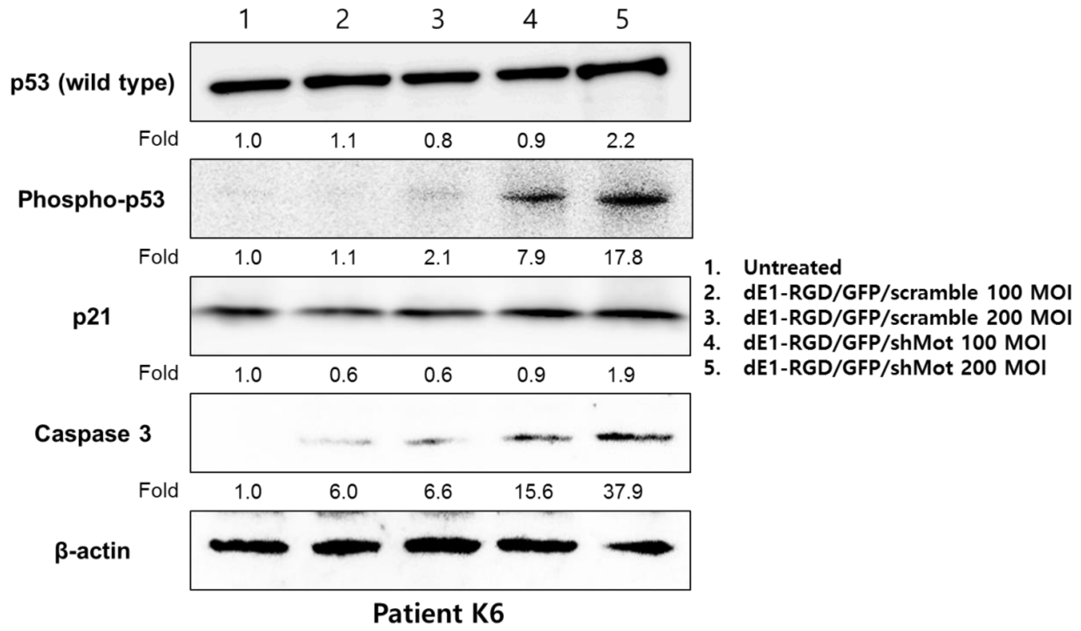

Supplementary Figure S3. Nuclear p53 expression level in primary keloid fibroblast was analyzed by western blotting at multiple time points following transduction with either dE1-RGD/GFP/scramble or dE1-RGD/GFP/shMot (200 MOI).

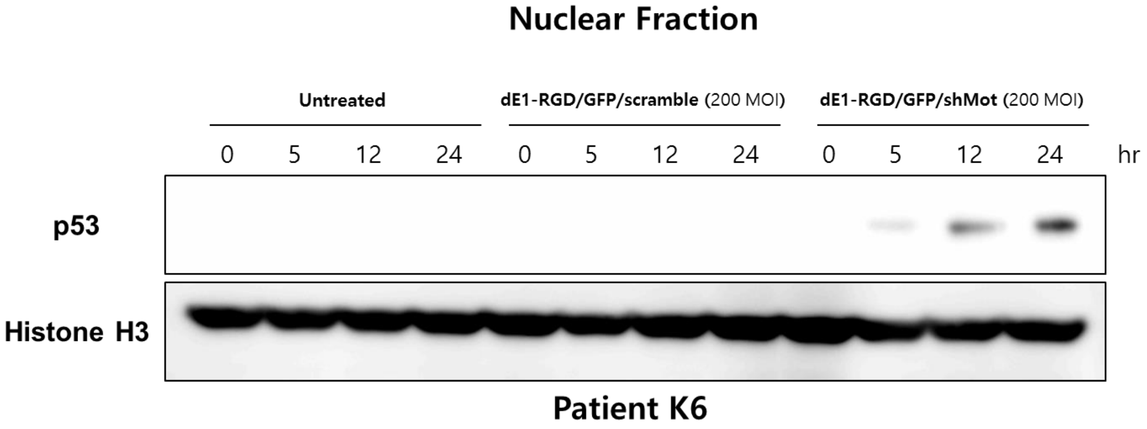

Supplement: Supplementary file 1 — Supplementary Figure Legends [file 41598_2017_13485_MOESM1_ESM.pdf]
